# Focal pulmonary interstitial opacities adjacent to the thoracic spine osteophytes among the cases with right-sided aortic arch

**DOI:** 10.1186/s40064-015-1212-3

**Published:** 2015-08-13

**Authors:** Rika Yoshida, Takashi Katsube, Takeshi Yoshizako, Hajime Kitagaki

**Affiliations:** Department of Radiology, Faculty of Medicine, Shimane University, 89-1, Enya-cho, Izumo, Shimane 693-8501 Japan

**Keywords:** Right-sided aortic arch, Focal pulmonary interstitial opacity, Thoracic spine osteophyte

## Abstract

**Objective:**

The purpose of this study was to evaluate the factors to development of focal pulmonary interstitial opacities adjacent to thoracic spine osteophytes among cases with right-sided aortic arch.

**Materials and methods:**

This was a retrospective review of our hospital information system on patients with right-sided aortic arch who underwent chest computed tomography (CT) from April 2003 to September 2014. CT were reviewed to evaluate the position and thickness of osteophytes and that of focal pulmonary opacities adjacent to osteophytes, comparing data between the patients with osteophytes with pulmonary opacity (group A) and patients with osteophytes without pulmonary opacity (group B).

**Results:**

There were 25 patients totally, 23 cases of left-sided thoracic osteophytes, two cases on both sides. Comparing Group A (n = 10) and Group B (n = 15), the presence of pulmonary opacities was significantly associated with thickness of osteophytes (Mann–Whitney U test*, P* < 0.05).

**Conclusion:**

In patients with right-sided aortic arch, thoracic osteophytes were often observed on the left side. The presence of pulmonary opacities adjacent to thoracic osteophytes was associated with thickness of osteophytes. Furthermore, these interstitial opacities should not be considered a preclinical form of fibrosing lung disease.

## Background

Osteophyte formation on the vertebral body is a very common age-related change In total, 80 % of men and more than 60 % of women 50 years of age or older are said to have this change (Nathan 1962; Resnick and Niwayama 1995; Klaassen et al. 2011; Wu and Shepard 2011). Moreover, thoracic osteophytosis did not occur in the area adjacent to the aorta on a chest CT (Goldberg and Carter 1978).

Otake et al. (2002) reported that the osteophytes of the thoracic vertebrae appeared to cause focal fibrosis in the subpleural region of the lower lobe of the right lung. In addition, they speculated that focal fibrosis may form in the left lung if the osteophyte developed on the left side secondary to the rightward elongation of the descending aorta (Otake et al. 2002).

In this study, we aimed to confirm whether thoracic osteophytes were observed on the left side anterior to the vertebrae on chest CT, as well as to evaluate factors involved in the development of focal pulmonary interstitial opacities adjacent to thoracic spine osteophytes among cases with right-sided aortic arch, where the descending aorta is located anterior to the thoracic vertebrae on the right.

## Methods

### Patients

**Fig. 1:**
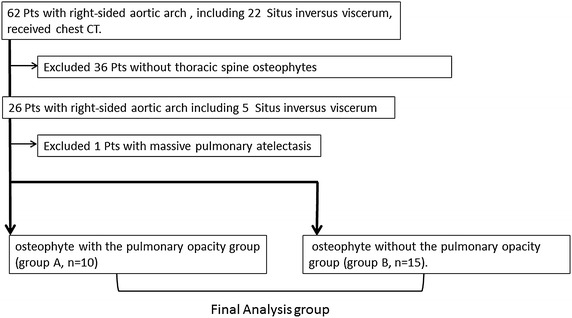
Flow chart of patient selection process.

We retrospectively searched the hospital and radiology information system of our hospital for patients diagnosed with right-sided aortic arch, including *situs inversus viscerum*, who underwent chest CT from April 2003 to September 2014. We excluded patients who did not have thoracic spine osteophytes (Fig. 1). Our Institutional Review Board approved this study and waived the need for informed consent from the patients.

### CT technique

Chest CT was performed with various scanners as follows, GE CT9800 (GE Healthcare America), SIEMENS SOMATOM PLUS40 (Siemens Medical Solutions, Forch- heim, Germany), PHILIPS Brilliance CT40, PHILIPS Brilliance CT64 (Philips Healthcare, Netherlands), TOSHIBA Aquilion 16, TOSHIBA Aquilion ONE, TOSHIBA Aquilion CX (Toshiba, Tokyo, Japan). Images of the entire lung were produced with varying section thickness, depending on time period it was performed: 10 mm, 2003–2004; 7 mm, 2003–2004; and 5 mm, 2006 onward. Lung window images were reconstructed with high-frequency algorithm (width, 1,600 HU; level, −600 HU).

### Image interpretation

**Fig. 2:**
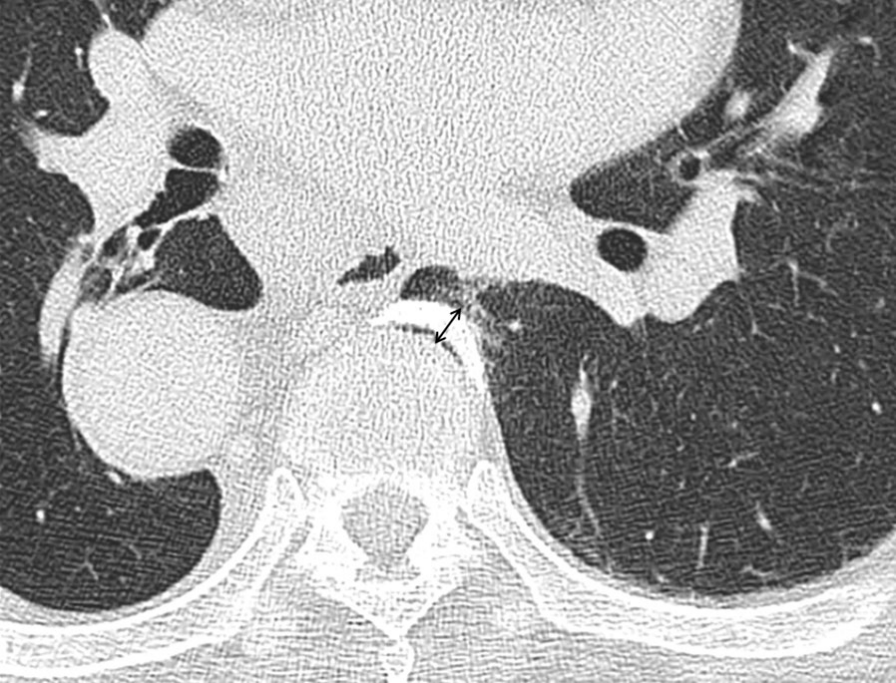
Method of osteophyte measurement. A 74-year-old man with a left-sided thoracic osteophyte associated with pulmonary opacity. Cumulative thickness of the osteophyte (*two-headed arrow*) and pulmonary opacity (reticular type) was 6 mm.

Two radiologists, one with 12 years’ experience and the other with 15 years’ experience in reading chest CT scans, independently reviewed the reviewed CT scans and evaluated the following factors: position of thoracic osteophytes relative to the midline of the vertebral body (left, right, and both sides), thickness of thoracic osteophytes (Fig. 2), and presence or absence of focal pulmonary opacities adjacent to the osteophytes. We evaluated the only localized lung shadow in contact with the osteophytes, in order to exclude the dependent opacities in the posterior region of the lung on supine CT, by confirming that there was no abnormal shadow in the subpleural region except for the site of osteophytes.

For patients with several thoracic osteophytes, the thickest osteophyte adjacent to the lung field was chosen as representative.

Focal pulmonary opacities were evaluated for thickness and categorized according to the morphological type, reticular or linear shadow. The reticular shadow was defined as fine netlike arrangement of interstitial thickening. The linear shadow was defined as an elongated thin or thick line parallel to the pleura. We adopted the type of shadow which accounted for more than half when both reticular and linear patterns were seen on CT. Disagreement between the two observers was resolved by consensus.

Depending on the presence or absence of lung shadows, we divided osteophytes into two groups: those with pulmonary opacity (Group A) and those without pulmonary opacity (Group B).

### Statistical analysis

The abovementioned variables were compared between the two groups using Mann–Whitney U test for numeric values and Chi square test or Fisher’s exact test for categorical values. A *P* value of <0.05 was considered statistically significant. Statistical analysis was performed using Statistical Package for the Social Sciences (SPSS), version 22.0 for Windows (IBM, Japan).

## Results

### Clinical findings

In total, this study included 25 patients 15 male, 10 female) with a median age of 66 years (range 36–82 years; Table 1). There were five patients with *situs inversus viscerum*. Focal interstitial opacities in the subpleural region of the left lower lobe that were adjacent to osteophytes were observed in 10 cases (40 %; Group A).

**Table 1:** **Patients characteristics and CT findings between Group A and Group B**

	Group A	Group B	*P*-value
Number of cases	10	15	
Sex (male: female)	5:5	10:5	
Age (mean ±SD), (range)	70.7 ± 6.9 (55–77)	63.9 ± 7.8 (39–82)	
Osteophyte’s position (left: both)	9:1	14:1	
Osteophyte’s thickness (mean ± SD), (range)	8.1 ± 1.8 (6–11)	5.8 ± 2.7 (3–13.5)	*
Pulmonary opacity’s thickness (mean, range)	4.5 (2–8)		
Pulmonary opacity’s shape (reticular: linear)	4:6		

Group A osteophyte with the pulmonary opacity group (n=10), Group B osteophyte without the pulmonary opacity group (n=15), Mann–Whitney U test was used.

* *P* values < 0.05 was considered significant.

During the follow-up period, chest CT scans were performed 1–11 times for each patient (average, 3.5 times).

### CT findings

Table 1 shows a comparison of CT findings between Group A and Group B. In total, thoracic osteophytes were located on the left in 23 cases (92 %) and on both sides anterior to the vertebra in two cases (8 %). In Group A (n = 10), pulmonary opacities were observed in the left lung, opposite side to the thoracic descending aorta. Between the two groups, the presence of pulmonary opacities was significantly associated with osteophyte thicknesses (*P* < 0.05). There was no significant difference between groups in terms of age and sex distribution (*P* > 0.05). The representative examples are shown in Fig. 2.

In Group A, five patients underwent chest CT several times. Their interstitial pulmonary opacities in contact with osteophyte were reproducible in cases that chest CT had taken more than once.

## Discussion

Osteophyte formation of the vertebral body is a very common change occurring with age (Nathan 1962; Resnick and Niwayama 1995; Klaassen et al. 2011; Wu and Shepard 2011).

A right-sided aortic arch is a rare congenital abnormality observed in 0.01 % of adults. Regardless of its rare occurrence, we occasionally encounter it in clinical practice (Bialowas et al. 2000; Kimura-Hayama et al. 2010; Salanitri 2005; Kawano et al. 2010; Hara et al. 2001; Kleinman et al. 1994; Glew and Hartnell 1991; Edwards 1948).

Goldberg et al. reported that no osteophyte was noted on the site in contact with the aorta on CT (Goldberg and Carter 1978), due to the preventive effect of aortic pulsations on osteophyte formation in the thoracic spine (Culver and Pirson 1960). Considering the technology and spatial resolution of CT at that time (1978), small osteophytes were expected not to be visualized. Nevertheless, their results showed a tendency for thoracic osteophytes to be observed on the left side anterior to the vertebrae among patients with descending aorta located anterior to the thoracic spine on the right, and supported the previous result reported by Nathan et al. (Nathan and Schwartz 1962).

In this study on patients with right-sided aortic arch, in which the thoracic descending aorta travels along the right ventral side of the vertebral body, most osteophytes were found on the left side of the lower thoracic vertebrae. Our study result supports previous reports (Goldberg and Carter 1978; Otake et al. 2002) in addition to the two cases that presented with osteophytes on both sides of the vertebra.

Consequently, focal pulmonary opacities were noted in the left lung. Limited abnormalities were reported by Otake et al. including that the pulmonary interstitial opacity adjacent to the thoracic osteophytes do not appear to progress and should not be considered a preclinical form of more extensive fibrosing lung disease (Hansell 2010). The phenomenon of fibrosis adjacent to spinal osteophytes in older individuals is seen as localized ground-glass opacity or a reticular pattern adjacent to osteoarthritis protrusions and is easily recognized (Hansell 2010). The pulmonary opacities in our results were highly-reproducible and did not progress on follow-up, similar to the findings of Otake et al. (2002).

Otake et al. showed that the histological findings of the focal pulmonary opacity were fibrosis; these irreversible findings may have been due to compression of the osteophytes and did not depend on position (Otake et al. 2002). Among cases with right-sided aortic arch, the focal pulmonary opacities adjacent to the osteophytes would be expected to have the same histological findings. These reports signify that pulmonary interstitial opacities adjacent to osteophytes could be one of the most famous pseudo lesions that radiologists need to know when evaluating chest CT. The experienced radiologists will not misdiagnose the focal pulmonary opacities in contact with the osteophytes as pulmonary interstitial pneumonia. However, in some cases, it may be difficult to differentiate the focal pulmonary interstitial opacities adjacent to thoracic spine osteophytes from the peripheral-type lung cancer.

For cases with right aortic arch and descending aorta located anterior to the thoracic spine, it will be necessary to observe the left lung for pulmonary opacities adjacent to osteophytes. Likewise, the association of osteophyte thickness with the development of pulmonary opacities adjacent to thoracic spine osteophytes is similar to a previous report (Otake et al. 2002).

This study has some limitations. First is the small number of cases with right-sided aortic arch and *situs inversus viscerum* owing to their rare occurrence. Second is the long study period which encompassed different ways of chest CT evaluation, model, and display conditions. Therefore, future prospective studies considering these biases are needed.

## Conclusion

Among cases with right-sided aortic arch, most thoracic osteophytes were observed on the left side anterior to the vertebrae. The presence of pulmonary opacities adjacent to thoracic spine osteophytes was associated with osteophyte thickness. Furthermore, focal pulmonary interstitial opacities should not be considered a preclinical form of fibrosing lung disease.

## Abbreviations

CT: computed tomography
SPSS: statistical package for the social sciences
